# Identification of novel *L2HGDH* mutation in a large consanguineous Pakistani family- a case report

**DOI:** 10.1186/s12881-018-0532-x

**Published:** 2018-02-20

**Authors:** Muhammad Ikram Ullah, Abdul Nasir, Arsalan Ahmad, Gaurav Vijay Harlalka, Wasim Ahmad, Muhammad Jawad Hassan, Emma L. Baple, Andrew H. Crosby, Barry A. Chioza

**Affiliations:** 1 Department of Biochemistry, Faculty of Biological Sciences, Quaid-i-Azam University, Islamabad, Pakistan; 2 RILD Wellcome Wolfson Centre - Level 4, Royal Devon and Exeter NHS Foundation Trust, University of Exeter Medical School, Barrack Road, Exeter, EX2 5DW UK; 3 Computational Medicinal Chemistry Laboratory, Department of Biochemistry, Abdul Wali Khan University Mardan, Mardan, Pakistan; 4 Division of Neurology, Shifa International Hospital, Shifa Tameer e Millat University, Islamabad, Pakistan; 5 Department of Healthcare Biotechnology, Atta-ur-Rahman School of Applied Biosciences (ASAB), National University of Sciences & Technology (NUST), Islamabad, Pakistan

**Keywords:** *L2HGDH*, Mutation, Pakistan, L-2-hydroxyglutaric aciduria, Cerebellar ataxia, Epilepsy, Developmental delay, Intellectual disability

## Abstract

**Background:**

L-2-hydroxyglutaric aciduria (L2HGA) is a progressive neurometabolic disease of brain caused by mutations of in L-2-hydroxyglutarate dehydrogenase (*L2HGDH*) gene. Cardinal clinical features include cerebellar ataxia, epilepsy, neurodevelopmental delay, intellectual disability, and other clinical neurological deficits.

**Case presentation:**

We describe an index case of the family presented with generalised tonic-clonic seizure, developmental delay, intellectual disability, and ataxia. Initially, the differential diagnosis was difficult to be established and a SNP genome wide scan identified the candidate region on chromosome 14q22.1. DNA sequencing showed a novel homozygous mutation in the candidate gene *L2HGDH* (NM_024884.2: c.178G > A; p.Gly60Arg). The mutation p.Gly60Arg lies in the highly conserved FAD/NAD(P)-binding domain of this mitochondrial enzyme, predicted to disturb enzymatic function.

**Conclusions:**

The combination of homozygosity mapping and DNA sequencing identified a novel mutation in Pakistani family with variable clinical features. This is second report of a mutation in *L2HGDH* gene from Pakistan and the largest family with L2HGA reported to date.

## Background

L-2-hydroxyglutaric aciduria (L2HGA) is a rare autosomal recessive neurodegenerative disorder of metabolism [OMIM #236792] which is due to the accumulation of L-2-hydroxyglutaric acid (LGA) in urine, plasma and cerebrospinal fluid (CSF) [1, 2]. The phenotypic features of this organic aciduria are diverse, including developmental delay, cerebellar ataxia, epilepsy, severe intellectual disability, and macrocephaly [3–5]. The onset of disease has been reported to occur at an early age with severe epileptic fits or neurodegenerative symptoms, although it may also appear in adulthood with less severe presentations. There are reports of increased incidence of the development of brain tumours due to progression in L2HGA [6–9].

The diagnosis of L2HGA comprises of biochemical, radiological and genetic testing. The MRI abnormalities seen in the subcortical cerebral white matter, putamen, caudate nucleus, globus pallidus, and dentate nucleus are unique to L2HGA, and are used as baseline investigation [6, 10–16]. The disease-causing gene is L-2-hydroxyglutarate dehydrogenase (*L2HGDH*-NM_024884.2) which is located on chromosome 14q22.1 [MIM 609584] and comprises of 10 coding exons spanning 75 kb. It is expressed in various tissues with the highest expression found in the brain [15, 17]. The gene encodes a protein of 463 amino acids, specifying a mitochondrial targeting sequence (aa 1-50) and a domain for family of FAD-dependent enzymes [15]. L2HGDH is a mitochondrial enzyme which catalyses oxidation of L-2-hydroxyglutarate (L2HG) to α2-ketoglutarate (α2KG); a metabolic product bound to mitochondrial membrane [15, 17]. Several mutations *L2HGDH* have been reported worldwide in affected individuals belonging to various ethnic groups [6, 8, 13–16, 18, 19] (http://grenada.lumc.nl/LOVD2/vumc/status.php) [17].

The present case describes the clinical presentation and mutation analysis of *L2HGDH* in a large Pakistani consanguineous family comprising multiple individuals affected by a metabolic neurological disorder. Homozygosity and sequencing studies revealed a rare missense mutation (NM_024884.2:c.178G > A; p.Gly60Arg), in exon 2 of *L2HGDH* as the likely cause of disease in this family.

## Case presentation

A 16 year old girl (IV-4) presented to hospital with history of seizures since the age of 8 months, intellectual delay, and ataxia. At the age of 13 years she was described by parents as ‘mentally dull’ and generalized tonic-clonic seizures recurred with increased frequency, mostly at night. Her elder sister (IV-6) and brother (IV-1) also showed symptoms of epilepsy and intellectual delay, as did three first cousins (IV-8, IV-9, and IV-10) although no in depth clinical evaluation was performed.

### Clinical evaluation of index case

**Fig. 1:**
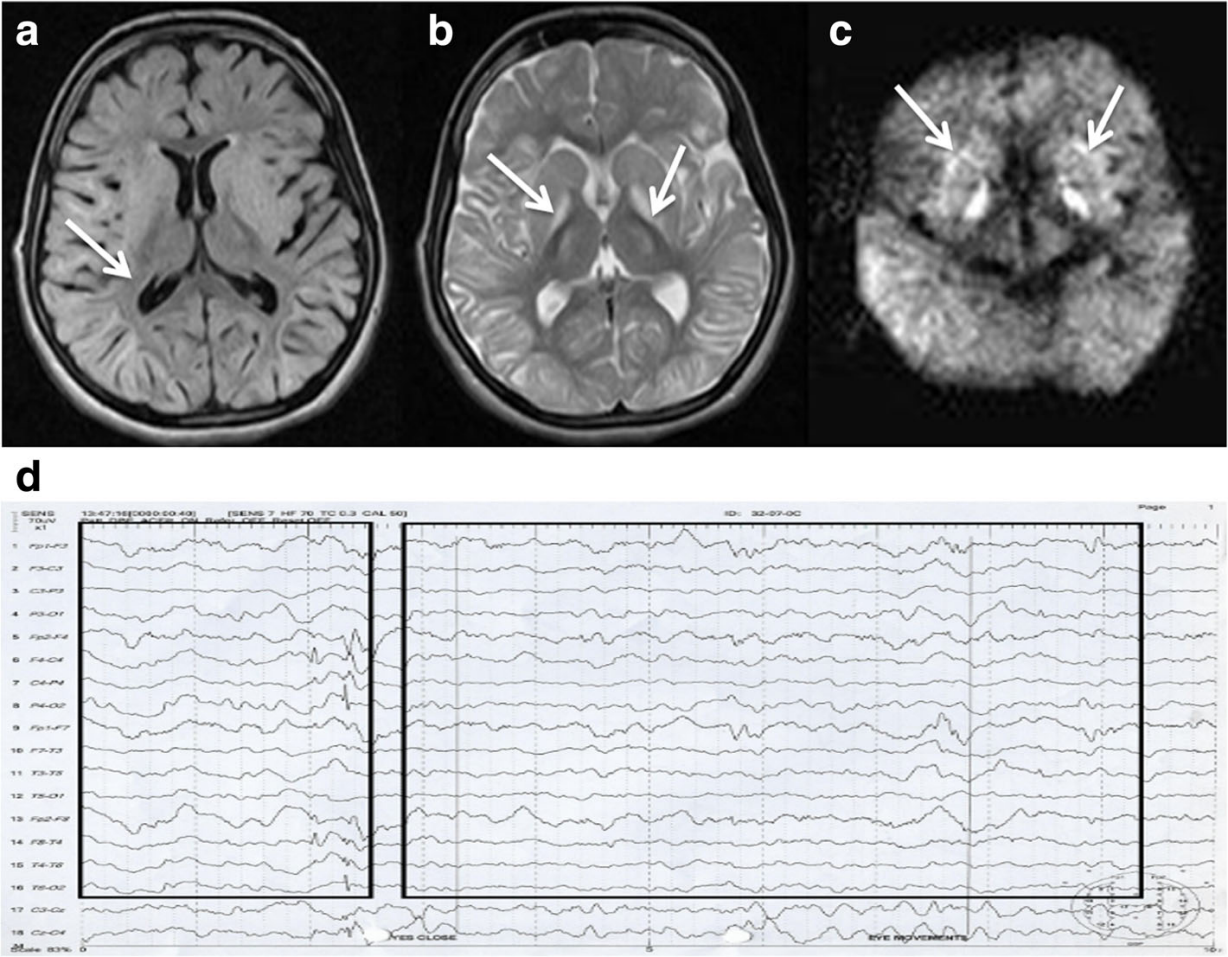
Clinical features of individuals homozygous for *L2HGDH* c.178G > A. Patient VI:4 at 16 years of age, showed diffuse T2 hyperintense signals abnormality in the subcortical white matter (**a**), bilateral symmetrical T2 hyperintense signals in bilateral basal ganglia (**b**) and cerebellar atrophy (**c**) and epileptiform changes in EEG (**d**)

On physical and clinical examination IV-4 was alert, with an ataxic gait. Manual muscular testing did not note any weakness of limbs, but mild finger and nose ataxia was apparent along with a retarded capability of speech. Her deep tender reflexes were +++ and symmetrically preserved, while the plantar responses were bilaterally flexor. Her brain MRI showed abnormal diffuse T2 hyperintense signals in the subcortical white matter and bilateral symmetrical T2 hyperintense signals in bilateral basal ganglia (Fig. 1). Mild cortical cerebellar atrophy was also seen. Electroencephalogram (EEG) examination showed moderate diffuse encephalopathy/moderate diffuse brain dysfunction and observed epileptiform activity arising from the right hemisphere (Fig. 1). Urine testing for L2-hydroxyglutarate was not possible due to unavailability of this test in regional diagnostic laboratories, and remote setting of the family involved. Genetic investigation was recommended and she was advised for tablet Neurobion 1 BD, tablet Folic acid 5 mg OD, capsule Coenzyme Q-10, 50 mg BD, tablet Loprin 75 mg for symptomatic management.

### Molecular genetic analysis

Genomic DNA was extracted from peripheral blood samples. Due to the availability of multiple affected family members and the consanguinity of the parents of the index case (Fig. 1a), autozygosity mapping through genome-wide SNP genotyping was conducted as previously described using Illumina Human CytoSNP-12 v2.1 microarrays [20]. A 24.5 Mb homozygous region on chromosome 14 (chr14:36,976,285-61,626,155 [hg38]) shared among all affected individuals delimited by recombinant SNP markers rs10483479 and rs2244057. The disease locus was predicted to contain 327 genes including *L2HGDH* [MIM: 236,792], previously implicated in causing overlapping neurological phenotypes. Subsequent di-deoxy sequence analysis of exon 2 (amplicon size 891 bp; primers: 5’-CCAATACATTGCTCTGTCGC-3′; 5’-AAAGTGAGCACAATCCTGGG-3′; Cycling conditions: Denaturation at 95 °C for 2 min; 2 cycles of 30 s at 95 °C, 30 s at 66 °C, 30 s at 72 °C; 2 cycles of 30 s at 95 °C, 30 s at 64 °C, 30 s at 72 °C; 35 cycles of 30 s at 95 °C, 30 s at 62 °C, 30 s at 72 °C and a final extension of 2 min at 72 °C) revealed a rare/novel missense variant with a single heterozygous individual of South Asian origin reported in 60,680 samples by the ExAC Consortium (http://exac.broadinstitute.org/variant/14-50769698-C-T; rs771556952; NM_024884.2:c.178G > A; p.Gly60Arg), which was found to co-segregate in the extended pedigree. The variant was not present in 150 chromosomes of Pakistani ancestry.

### Computational analysis

**Fig. 2:**
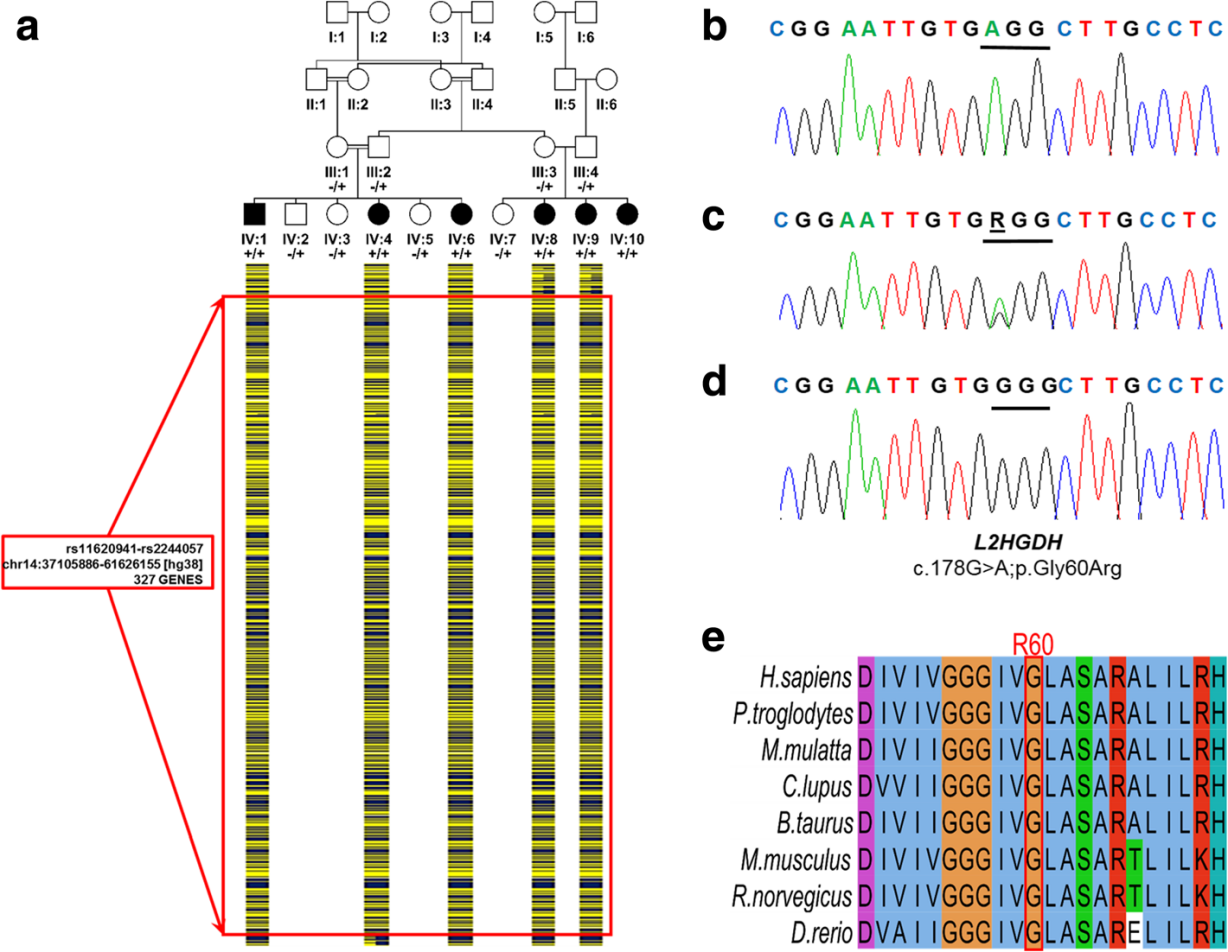
Family pedigree showing *L2HGDH* c.178G > A genotype data and images of affected individuals. **a** Simplified pedigree of the extended Pakistani family investigated, with pictorial representation of genotypes across ∼24 Mb of chromosome 14 encompassing the disease locus (dashed blue boxed region, red boxed region). All affected individuals were subsequently shown to be homozygous for the *L2HGDH* variant NM_024884.2:c.178G > A (indicated). Parental samples were heterozygous, and unaffected siblings were either WT or heterozygous carriers. **b-d** Electropherograms showing the DNA sequence at the position of *L2HGDH* c.178G > A in a homozygous affected (**b**), heterozygous father (**c**) and WT control (**d**) and amino acid alignment using ClustalW showing high conservation of the G60 residue across vertebrates (**e**)

Amino acid sequence alignment using the program ClustalW 2.1 showed high conservation of the Gly60 residue in related vertebrates (Fig. 2). The variant alters a stringently conserved amino acid residue and is predicted to be highly damaging by standard prediction programs using the Emsembl Variant Effect Predictor web interface (FATHMM score = − 6.21 (Damaging); MutationTaster score = 1 (Disease causing); PolyPhen2 score (HumDiv) = 1 (Probably damaging); PolyPhen2 score (HumVar) = 1 (Probably damaging); PROVEAN score = − 7.46 (Damaging); SIFT score = 0 (Deleterious)).

**Fig. 3:**
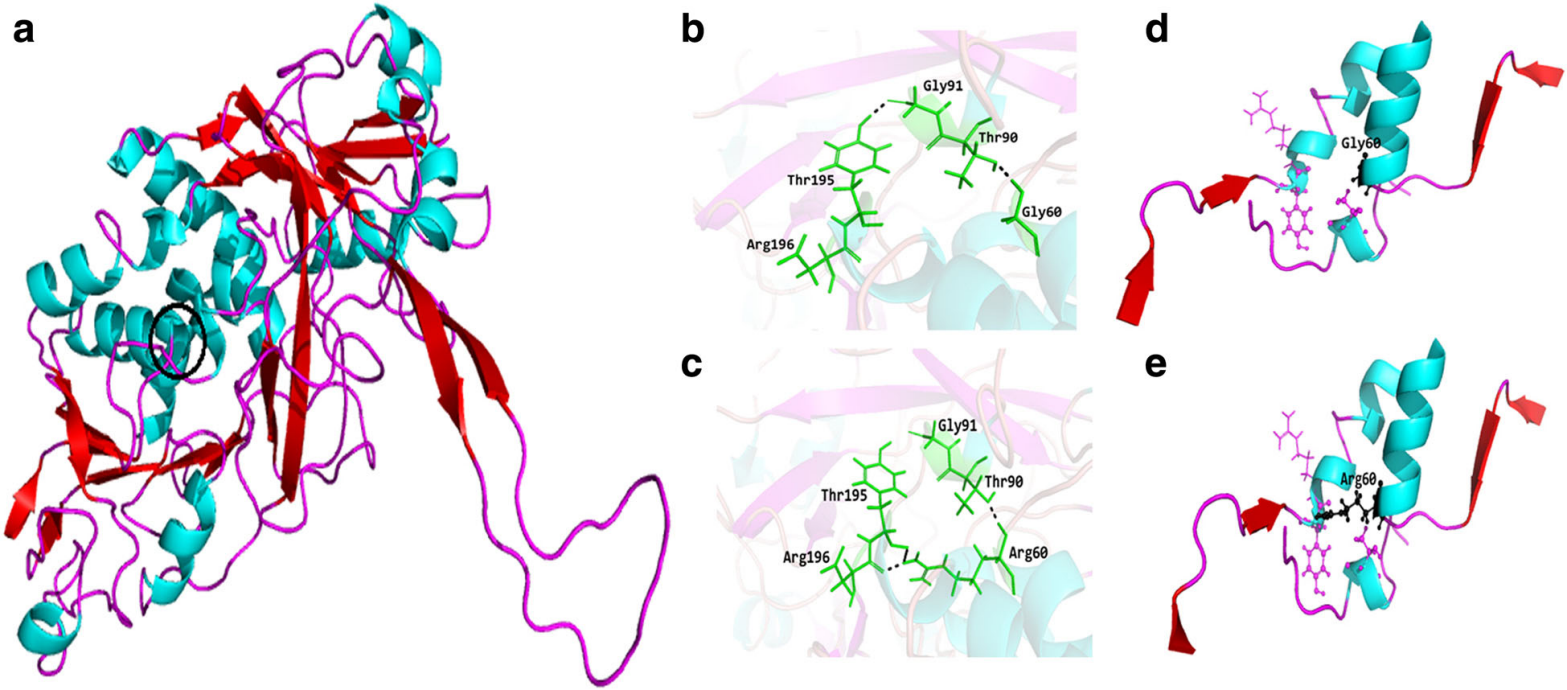
**a** Representation of predicted structure for human L2HGDH by means of Molecular Operating Environment (MOE v2013) software package, computationally predicted mutation is highlighted by circle (**b**) Representation of wild type protein interactions (**c**) Mutant type protein interactions (**d**) Secondary structure pattern of predicted wild and (**e**) Mutant type protein of human L2HGDH

On structural analysis, the Gly60 residue of L2HGDH resides in the helix region. This short non-polar glycine residue is replaced in the mutant protein by the larger, more positively charged and hydrophilic arginine residue. The Gly60Arg mutation is predicted to produce a minor local conformational change due to the difference in the observed contacts and surface area. The native glycine residue is only involved in intermolecular interactions with threonine at position 90 while the replaced basic arginine introduces an electrically charged, basic guanidium group which, unlike glycine, has more hydrogen bonding capabilities leading to the formation of an a inter molecular hydrogen bond with Thr90 as well as with Arg196 and Thr195. This leads to a slight local perturbation of the helix conformation for mutated protein (Fig. 3).

## Discussion and conclusions

In this study, we describe a large pedigree from Pakistan showing multiple neurological symptoms. Homozygosity mapping and Sanger sequencing revealed a novel missense mutation in *L2HGDH* gene. This is the second report of *L2HGDH* mutation in a Pakistani family; previously a nonsense mutation (p.Arg335Ter) was reported in a family showing a neuro-degenerative disorder of metabolism with two affected individuals [8]. Clinical and radiologic examinations of affected individuals identified presence of a slowly progressive neurodegenerative disease with cerebellar ataxia, seizures, delay in growth and abnormal subcortical white matter. MRI showed the persistent changes in the subcortical white matter characteristic in L2HGA leukoencephalopathy while the brain stem involvement in other leukoencephalopathy [11, 15]. Although additional phenotypic characteristics are described in the literature, including macrocephaly, pyramidal and extra-pyramidal features, these were not present in our patients, [15, 21].

*L2HGDH* encodes L-2 hydroxyglutare dehydrogenase which is the key contributor for this neurodegenerative disease. A large number of families and cases are reported with more than 100 pathogenic mutations in this gene. These mutations are mostly repeated in different ethnic populations. Interestingly, the disease is mostly reported in families from Mediterranean origin with numerous families reported from Turkey, Tunisia, Italy and Lebanon [6, 8, 15, 17]. Currently, there are 162 families with 283 cases which have been investigated for mutations in *L2HGDH* gene comprising a total of 112 mutations, 36 of which are found repeatedly found in different ethnic groups (http://grenada.lumc.nl/LOVD2/vumc/status.php).

The possible impact of our mutation on protein function was investigated using in silico bioinformatics tools. The mutation was predicted to affect the hydrogen bonding, and thus alter the stereochemistry of the protein (Fig. 3) [22].

To conclude, this case report provides the molecular diagnosis of a large consanguineous Pakistani family with six individuals. We identified a novel *L2HGDH* mutation predicted to cause in a loss of stability of L2HGDH protein.

## Abbreviations

aa: Amino acid
Arg: (amino acid symbol) Arginine
CSF: Cerebrospinal fluid
Glu: (amino acid symbol) Glutamic Acid
Gly: (amino acid symbol) Glycine
L2HG: L-2-hydroxyglutarate
L2HGA: L-2-hydroxyglutaric aciduria
*L2HGDH*: (gene symbol) L-2-Hydroxyglutarate Dehydrogenase
L2HGDH: (protein symbol)L-2-Hydroxyglutarate Dehydrogenase
Leu: (amino acid symbol) Leucine
LGA: L-2-hydroxyglutaric acid
Ser: (amino acid symbol) Serine
Thr: (amino acid symbol) Threonine
α2KG: α2-ketoglutarate
